# Allograft of Sertoli Cell Transplantation in Combination with Memantine Alleviates Ischemia-Induced Tissue Damages in An Animal Model of Rat

**DOI:** 10.22074/cellj.2020.6689

**Published:** 2019-12-15

**Authors:** Zeinab SafialHosseini, Mohammadreza Bigdeli, Sepideh Khaksar, Abbas Aliaghaei

**Affiliations:** 1 Department of Physiology, Faculty of Life Sciences and Biotechnology, Shahid Beheshti University, Tehran, Iran; 2 Institute for Cognitive and Brain Science, Shahid Beheshti University, Tehran, Iran; 3 Department of Herbal Science, Faculty of Biological Sciences, Alzahra University, Tehran, Iran; 4 Department of Anatomy and Cell Biology, School of Medicine, Shahid Beheshti University of Medical Science, Tehran, Iran

**Keywords:** Brain Ischemia, Cell Transplantation, Memantine, Sertoli Cell

## Abstract

**Objective:**

Brain ischemia is the most common disease in the world caused by the disruption of the blood supply of brain tissue. Cell therapy is one of the new and effective strategies used for the prevention of brain damages. Sertoli cells (SCs) can hide from the host immune system and secrete trophic factors. So, these cells have attracted the attention of researchers as a therapeutic option for the treatment of neurodegenerative diseases. Also, memantine, as a reducer of glutamate and intracellular calcium, is a suitable candidate for the treatment of cerebral ischemia. The principal target of this research was to examine the effect of SC transplantation along with memantine on ischemic injuries.

**Materials and Methods:**

In this experimental research, male rats were classified into five groups: sham, control, SC transplant recipient, memantine-treated, and SCs- and memantine-treated groups. SCs were taken from another rat tissue and injected into the right striatum region. A week after stereotaxic surgery and SCs transplantation, memantine was injected. Administered doses were 1 mg/kg and 20 mg/kg at a 12-hour interval. One hour after the final injection, the surgical procedures for the induction of cerebral ischemia were performed. After 24 hours, some regions of the brain including the cortex, striatum, and Piriform cortex-amygdala (Pir-Amy) were isolated for the evaluation of neurological deficits, infarction volume, blood-brain barrier (BBB) permeability, and cerebral edema.

**Results:**

This study shows that a combination of SCs and memantine caused a significant decrease in neurological defects, infarction volume, the permeability of the blood-brain barrier, and edema in comparison with the control group.

**Conclusion:**

Probably, memantine and SCs transplantation reduce the damage of cerebral ischemia, through the secretion of growth factors, anti-inflammatory cytokines, and antioxidant factors.

## Introduction

Cerebral ischemia is the third reason of death and physical impairment in the world caused by the blood vessel blockage, through a blood clot or rupture of a vessel, responsible for the supply of a part of brain tissue (1). About 85% of stroke cases are caused by ischemia and 15% by a brain hemorrhage. The best way for controlling the stroke is the early prevention of ischemic damage expansion and thrombotic therapy (2). During cerebral ischemia, due to lack of oxygen and ATP, the ion pumps that are dependent on ATP, such as sodium-potassium and calcium pumps suffer from functional impairment (3). So, the excessive release of glutamate into the synaptic space leads to excitotoxicity.

Consequently, the extreme influx of extracellular calcium causes an imbalance in cellular homeostasis. An increase in the concentration of calcium inside the cell can activate the caspase enzymes that are in charge of inducing cell death and damages to ischemic cells. Moreover, intracellular calcium can increase the production of free radicals and cause more damages to ischemic cells (4). It should also be emphasized that the main reason for ischemic tissue damage is excitotoxicity. Therefore, a decrease in the concentration of glutamate in the synaptic space can significantly reduce ischemic damages (5).

One of the reasonable choice for reducing glutamate effects in synaptic space is blockage of the N-methyl-D aspartate (NMDA) receptor. NMDA is a receptor for stimulant neurotransmitters, called glutamate, which is a mediator of stimulant neural transmissions in the central nervous system. The excessive activity of this receptor leads to an increase in calcium intake, which provokes excitotoxicity and ultimately death of cells (6). In physiological conditions, the coupling of Mg^2+^ ion with the NMDA receptor prevents over-depolarization of the nerves, and in pathological conditions, lack of binding of Mg^2+^ ion to the NMDA receptor stimulates the nerve extremely (7). Memantine, as a non-competitive antagonist of the NMDA receptor, has a significant role in decreasing the destructive cytotoxicity of the stroke. Memantine is prescribed for the treatment of dementia, Alzheimer’s disease, and Parkinson’s disease. The advantage of this drug in comparison with other glutamate receptor antagonists is that it blocks the NMDA receptor without affecting on the natural activity of the receptor, leading to the reduction in neuronal function and excitotoxicity (8). This drug prevents the toxic interactions of free radicals, such as nitric oxide (NO) and reactivity oxygen groups (ROS) with vital macromolecules and also prevents the stimulation and activation of apoptosis-stimulating proteins, such as caspases, neural NO synthase (nNOS), and cytochrome C (9). In another report, it was proved that memantine alone and in combination with melatonin reduced brain damages due to the reduction of P38, ERK-1/2, and inducible NO synthase (iNOS) (10). Also, memantine ameliorated the pathogenesis of Alzheimer’s disease in animal models via blockage of the NMDA receptor and reduction of glutamate excitability (11).

In the brain, non-fatal ischemia can induce protective responses against subsequent intensive ischemic injury, called ischemic tolerance (12). The cerebral ischemia is common in people who are susceptible to cerebral ischemia, including patients with a history of heart attack and aneurysm. Accordingly, our purpose is the induction of ischemic tolerance by pretreatment of rats with Sertoli cells (SCs) and memantine.

Along with drug treatment, new strategies, such as cellbased therapy is used for the treatment of stroke. The most of cell resources, including fetal neural cells, stem cells, and SCs (as somatic cells) are suggested as an effective way for the management of some neurodegenerative diseases such as Alzheimer’s disease, amyotrophic lateral sclerosis, Huntington’s disease, and stroke. These cells are viable and can be replaced with the cells that reside in damaged tissues. They are also capable of reconstructing neural circuits and reducing functional impairment in the brain of patients with the above disorders (13). In the current research, transplanted SCs, as a pretreatment option, for brain ischemia were used. These cells are found in testicular tissues, possessing a high antigenic property, and providing a proper environment for the development of germ cells (14). Also, SCs express neurotrophic and growth factors, such as glial cell-derived neurotrophic factor (GDNF), insulin-like growth factor (IGF), transforming growth factor (TGF), vascular endothelial growth factor (VEGF), and fibroblast growth factor (FGF) (15). The reason for choosing the SCs for this research is the dominant properties of SCs compared with other somatic cells, including the inhibition of the immune system by SCs preventing the rejection of organ in a recipient.

Furthermore, SCs suppress the immune system by forming the tight connections around the nerve cells, thereby the production of interleukin-2 (IL-2) inhibitory factor, and inhibition of expression of major histocompatibility complex (MHC) (16). In another study, it was found that the co-culture of embryonic stem cells with SCs led to the differentiation of embryonic stem cells into dopaminergic neurons as a result of the presence of GDNF factor that is secreted from SCs and acts as a dopaminergic inducer in stem cells (17). Moreover, it was indicated that transplantation of SCs reduced the ischemic damages, such as infarction, brain edema, and the breakdown of the blood-brain barrier (BBB) (18). Concerning the characteristics mentioned about SCs, they could be applied as competent candidates for the amelioration of ischemic injuries.

In the current research, we focused on the effect of transplanted SCs in combination with the use of memantine in an animal model of cerebral ischemia.

## Materials and Methods

### Animal assignment and experimental protocol

In this experimental study, 98 adult male rats (with the weight range between 250 and 350 g, age range between 5 to 7 weeks) were procured from the Pasteur Institute. The water and food were given to all rats without restriction, and they were kept in standard conditions at the temperature of 22 ± 2˚C and 12:12 light-dark (LD) cycle. Rats were randomly divided into five groups: sham, control, allograft SC transplant recipient, memantinetreated group, and SC- and memantine-treated groups. The control group (n=21) consisted of rats that underwent ischemia following MCAO surgery. The control group was classified into three sub-groups to evaluate infarction (n=7), the BBB permeability (n=7), and brain edema (n=7). The sham group (n=14) consisted of rats that were suffered from the same surgery procedure of MCAO without importing suture and subdivided to assess the BBB permeability (n=7) and cerebral edema (n=7). The animals belonging to the allograft SC transplantation group received SCs from another rat tissue and were induced by MCAO surgery. The allograft SC transplantation group (n=21), the memantine-treated group (n=21), and the SC and memantine-treated group (n=21), divided into subgroups, as well as the control group. All rats were examined for the neurological deficit.

In this research, the two groups were selected for receiving SC culture medium and SCs without ischemic surgery. The comparison between these two groups and allograft SC transplantation did not indicate any significant difference. Also, the experimental methods were accomplished on vehicle (solvent of memantine) group. There was no remarkable difference between the vehicle group and other groups. So, the data of these groups are not shown.

### Ethical statement

This study was designed according to the rules of the National Institutes of Health and Care Guidance, the use of the Animal Laboratory (NIH Publications) revised in 2011, and the Ethics Committee of Shahid Beheshti University (No. 1012.667). Our whole effort was to use the lowest number of animals in this research.

### Pharmaceutical compound

Memantine, as a NMDA receptor blocker was used in this study was injected with different doses (1 mg/kg and 20 mg/kg; intraperitoneally). A week after stereotaxic surgery and SC transplantation, the injection of the drug was performed. Memantine was solved in saline. First, the high dose of 20 mg/kg and 12 hours later a low dose (1 mg/kg) was injected. One hour after the final injection, middle cerebral artery occlusion (MCAO) surgery was started. The minimum dose of memantine for each rat was 1 mg/kg, and the minimum toxic dose was 25 mg/ kg; so, the most appropriate dose used as the therapeutic dose was 20 mg/kg (19, 20). According to the previous studies, the dose of 20 mg/kg was selected in this research. By injecting this dose, the concentration of the drug in the brain tissue reaches 1-10 micromolar that this concentration is suitable for blocking a large number of NMDA receptors.

Moreover, the half-life of the drug is 12 hours. Therefore, for keeping the levels of the drug at 1-10 micromolar in the brain, 12 hours after the first injection, the preservative dose of 1 mg/kg was administered (19, 20).

### Isolation and culture of sertoli cell

At first, rats were sacrificed, and their testis removed and transferred into a falcon containing the culture medium and antibiotics. Under sterile conditions, the small pieces were separated from testicular tissue. Tissues were transmitted to tubes containing trypsin (0.25%, Gibco, USA) and were incubated at 37˚C for 15 minutes. First, the nephrotic tubes were separated; then, trypsin was aspirated, and 1% collagenase was added to the tubes consisting of tissues, and the samples were incubated at 37˚C for 15 minutes. After pipetting the samples and addition of the serum, the samples were centrifuged, and the resultant pellet was transferred into the culture flasks containing Dulbecco’s Modified Eagle’s Medium (DMEM/F12, Gibco, USA) culture medium supplemented with 10% fetal bovine serum (FBS, Gibco, USA) and antibiotics. After 48 hours of the culture period, the culture medium was replaced by the new one. When the cells reached to appropriate density, they were passaged with trypsin (18).

### Immunocytochemistry of Sertoli cells

SCs were transferred into the plates for 24 hours, and after the cells reached appropriate density, the whole culture medium was removed. Then, it was washed with phosphate buffered saline (PBS), and the cells were fixed by 4% paraformaldehyde solution. The cells were permeabilized by 0.3% Triton X-100 solution (Sigma Aldrich, USA), and then incubated in blocking solution. Afterward, the cells were incubated with the primary antibody (anti-GATA4, Abcam, USA) overnight. Subsequently, the cells were washed with PBS and incubated with the second antibody (goat conjugatedFITC anti-mouse antibody). The nuclei of the cells were stained by Hoechst and were observed under a fluorescent microscope. The presence of SCs was performed by the immunocytochemistry technique, as GATA4 was defined as a marker.

### Stereotaxic surgery and injecting Sertoli cells

After SCs reached to appropriate density, the cells were located in a suspension of a 2 μl DMEM aliquot. Then, they were separated with a trypsin solution. After centrifuging, the cells were counted with trypan blue staining method, and 500,000 cells were selected for the injection. Rats were anesthetized, and their heads were fixed in a stereotaxic device. After cleaning the skull surface, the distance between the Lambda and Bregma was found, and injection of the cells into the right striatum was carried out by a Hamilton micro-syringe, according to the coordinates of the brain atlas specified for rats: Bregma: +0.5 mm AP; ± 2.6 mm ML; -5 mm DV (21).

The injected cells were labeled with DiI (5 μg/ml for 20 minutes before the injection) (Sigma Aldrich, USA) and Hoechst staining. After 7 days, rats were sacrificed, and the brain was prepared for the determination of the survival and distribution of transplanted cells. The cells were detected by fluorescing microscopy.

### Induction of focal ischemic stroke

Anesthesia of rats was performed with chloral hydrate (400 mg/kg). According to the method explained by Lange et al. (22), MCAO surgery was performed. In this surgery, a 0-3 nylon suture was inserted to the internal artery through the trunk of the common carotid artery and to the anterior cerebral artery. It continued through the internal carotid artery. Due to the insertion of the suture and blockage of arterial blood flow, the blood flow was not able to reach MCA. After 60 minutes, the suture was withdrawn and the right hemisphere blood flow restored through the Willis Ring.

### Neurobehavioral evaluation

Behavioral evaluation of neurological defects was conducted 24 hours after the reperfusion process. The neurological finding was categorized into 5 parts, including motor function, sensory function, beam test, raise the Tail, and reflex activity. The maximum score for each animal was 18 (23).

### Infarction volume assessment

After 24 hours of the reperfusion process, rats were sacrificed with a high dose of an anesthetic drug, their brains were isolated, and kept at 4˚C for 10 minutes. Then, brains were coronally sectioned at a thickness of 2 µm. After that, 2% triphenyl tetrazolium chloride solution was poured on the slides, and incubated at 37˚C for 15 minutes. The summed infarct volume from all brain sections was calculated as the total infarct volume. The contribution of the edema to the infarct volume was modified with the formula mentioned below. The evaluation of infarct volume in the cortex, striatum, and the Piriform cortexamygdala regions was also accomplished separately (24). The corrected volume of damaged area=left hemisphere volume - (left hemisphere volume- damaged area volume).

### Assessment of brain edema

After removing the brains from the skulls, the weights of various areas including the striatum, cortex, and cortex Piriform-amygdala as the wet weight were measured and after incubation at 120˚C for 24 hours, the dry weight (DW) was also measured. Finally, the content of brain water was estimated according to the following formula (24). [(WW-DW)/WW]×100

### Measuring the permeability of the blood-brain barrier

After 30 minutes of the induction of ischemia, the injection of 2% Evans Blue solution (in the amount of 4 ml/kg) was carried out through the blood vein. Perfusion was performed using 250 ml saline, 24 hours after the withdrawal of suture. The different parts of the brain, including the striatum, cortex, and the Piriform cortex-amygdala were separated from each other, and each of them was weighed, separately. Then, it was homogenized with phosphate buffered solution, and after mixing with 60% acid trichloroacetic acid, it was agitated by a vortex for 3 minutes. Then, microtubes were kept at 4˚C for 30 minutes. In the next step, the samples were centrifuged at 1000 rpm for 30 minutes. Therefore, the light absorbance of brain solution was measured at the wavelength of 610 nm and compared with the standard concentration curve, and concentration of Evans Blue dry was calculated as μg/g of the brain tissue (24).

### Statistical analysis

All of the statistical analyses were conducted by the SPSS software version 22 (SPSS Inc, Chicago, IL, USA). The data from NDS were quantified by the nonparametric test (Kruskal-Wallis), followed by the Dunn test. The volume of tissue damage (infarction) was determined by the ImageJ software (Version 1.50), and the obtained findings were analyzed by one-way ANOVA. The comparison of the BBB integrity and brain edema were calculated by one-way ANOVA, followed by the Bonferroni post hoc test. The results were expressed as mean ± SEM. The level of significance was set at P<0.05.

## Results

### Confirming the presence of Sertoli cells in testiclederived cells

**Fig 1:**
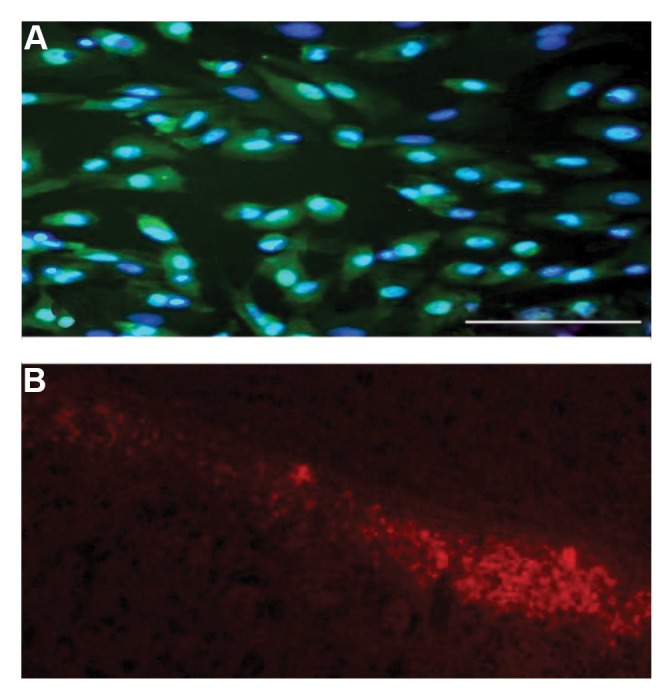
The identification and presence of Sertoli cells in testicle-derived cells. **A.** Sertoli cells expressed GATA4 marker that is shown in green. Staining the cultured cells with Hoechst showed that the Sertoli cells with blue nuclei (scale bar: 200 µm, ×20). The immunocytochemistry proves that the cultured cells are Sertoli cells. **B.** Staining the injected Sertoli cells with DiI. By using fluorescent microscopy, it was illustrated that the injected cells are alive after 7 days (scale bar: 200 µm, ×20).

The immunocytochemistry analysis of SCs against antiGATA4 antibody showed that the cultured cells expressed the GATA4 marker. GATA4 is expressed in SCs (green color). The nuclear staining conducted by Hoechst confirmed that the blue nuclei belonged to SCs (Fig .1A).

### Survival of injected cells in the striatum after 7 days

Staining injected SCs with DiI (a fluorescent lipophilic cationic indocarbocyanine dye) confirmed the survival of injected cells in the striatum after 7 days (Fig .1B).

### The effect of Sertoli cell transplantation and injection of memantine on neurological deficits

**Fig 2:**
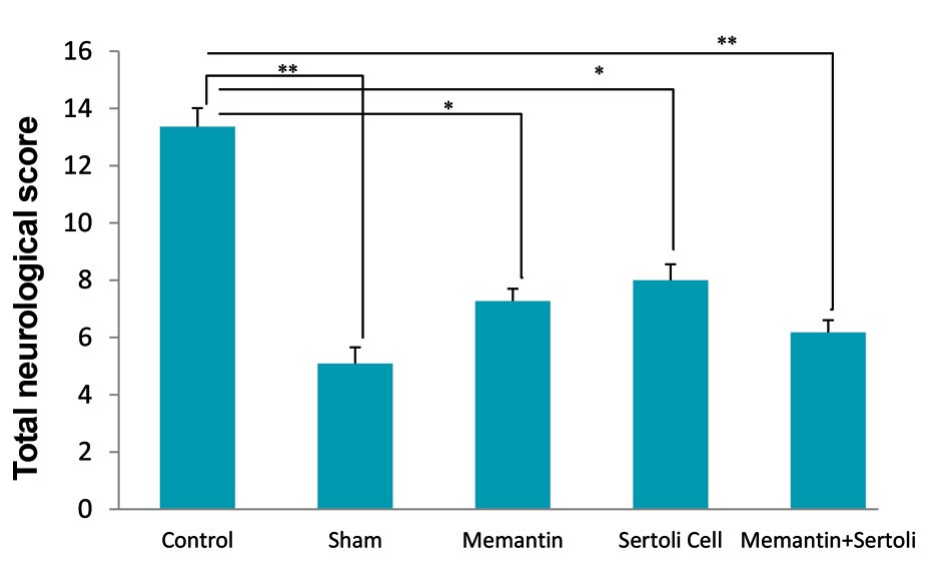
The effect of the Sertoli cell transplantation, memantine, and allograft transplantation of Sertoli cells plus memantine on total neurological deficit scores. Values are expressed as the mean ± SEM (n=10). P˂0.05 compared with the control group (Nonparametric KruskalWallis analysis). *; P<0.05 and **; P<0.01.

The analysis of the total score of the behavioral assessment showed that in the allograft SC transplantation, memantine, and allograft SC transplantation+memantine a significant decrease groups was observed in neurological deficits compared with the control group. Moreover, the total score obtained from the neurological examinations in the sham group had a significant decrease compared with the MCAO surgery or control group (Fig .2). The partial tests were statistically analyzed separately in the experimental groups. The results revealed that SCs, memantine, and the simultaneous administration SCs with memantine exerted a reduction in neurological tests such as reflex activity, sensory functions, and motor functions (Table 1).

**Table 1:** The partial neurologic deficit scores in each experimental group (n=10)


Experimental groups	Rats	Raise the tail	Motor function	Sensory function	Beam test	Reflex activity	Sum	Average	Survey result

Control (MCAO)	1	2	4	2	2	1	11	13.2	Meaningful to the sham group (P<0.001)RT: 1-2 (P=0.002)MF: 1-2 (P=0.001)SF: 1-2 (P=0.002)BT: 1-2 (P=0.003)RA: 1-2 (P=0.001)
	2	2	4	2	2	2	12		
	3	1	5	2	3	2	13		
	4	3	5	2	3	2	15		
	5	3	4	2	2	3	14		
	6	3	6	2	2	2	15		
	7	2	5	2	2	2	13		
	8	2	4	2	3	3	14		
	9	2	5	2	2	2	13		
	10	2	5	2	2	1	12		
Sham	1	1	1	2	1	1	6	5	
	2	0	2	1	0	1	4	
	3	0	2	0	2	2	6	
	4	0	2	1	1	0	4	
	5	1	3	1	0	1	6	
	6	0	1	1	2	0	4	
	7	0	1	2	1	1	5	
	8	0	1	1	1	1	4	
	9	1	2	1	1	0	5	
	10	0	1	1	2	2	6	
Sertoli cells(SCs cells)	1	0	3	2	1	1	7	8	Meaningful to the control group (P=0.04)RT: 3-1 (P=0.01)MF: 3-1 (P=0.03)SF: 3-1 (P=0.03)BT: 3-1 (P=0.05)RA : 3-1 (P=0.03)
	2	1	5	2	1	1	10		
	3	1	3	1	1	1	7		
	4	2	5	1	1	2	11		
	5	0	4	2	2	2	10		
	6	1	4	0	1	1	7		
	7	1	2	1	1	1	6		
	8	1	5	1	1	1	9		
	9	1	3	2	0	2	8		
	10	0	2	1	1	1	5		
Memantine	1	1	3	1	1	1	7	7.1	Meaningful to the control group (P=0.01)RT: 4-1 (P=0.03)MF: 4-1 (P=0.01)SF: 4-1 (P=0.03)BT: 4-1 (P=0.02)RA: 4-1 (P=0.001)
	2	2	2	1	1	2	8		
	3	1	3	2	0	1	7		
	4	0	5	1	1	1	8		
	5	1	4	1	0	1	7		
	6	1	3	2	0	0	6		
	7	1	3	1	1	1	7		
	8	1	4	1	1	1	8		
	9	0	3	1	2	1	7		
	10	1	2	2	0	1	6		
SCs+Memantine	1	2	2	1	2	2	9	6.1	Meaningful to the control group (P<0.001)RT: 5-1 (P=0.01)MF: 5-1 (P=0.001)SF: 5-1 (P=0.003)BT: 5-1 (P=0.04)RA: 5-1 (P=0.02)
	2	2	1	1	2	2	8		
	3	2	3	0	0	1	6		
	4	1	2	1	1	2	7		
	5	1	1	1	1	0	4		
	6	0	3	1	0	1	5		
	7	0	2	1	1	2	6		
	8	1	2	1	1	2	7		
	9	1	1	1	1	1	5		
	10	1	1	0	1	1	4		


MCAO; Middle Cerebral Artery Occlusion, SCs; Sertoli cells, RT; Raise the tail, MF; Motor function, SF; Sensory function, BT; Beam test, RA; Reflex action, 1: Control group, 2; Sham group, 3, Sertoli cell group, 4; Memantine group, and 5; The Sertoli cell transplantation+memantine (Nonparametric KruskalWallis analysis).

### The effect of Sertoli cell transplantation and injection of memantine on infarct volume

**Fig 3:**
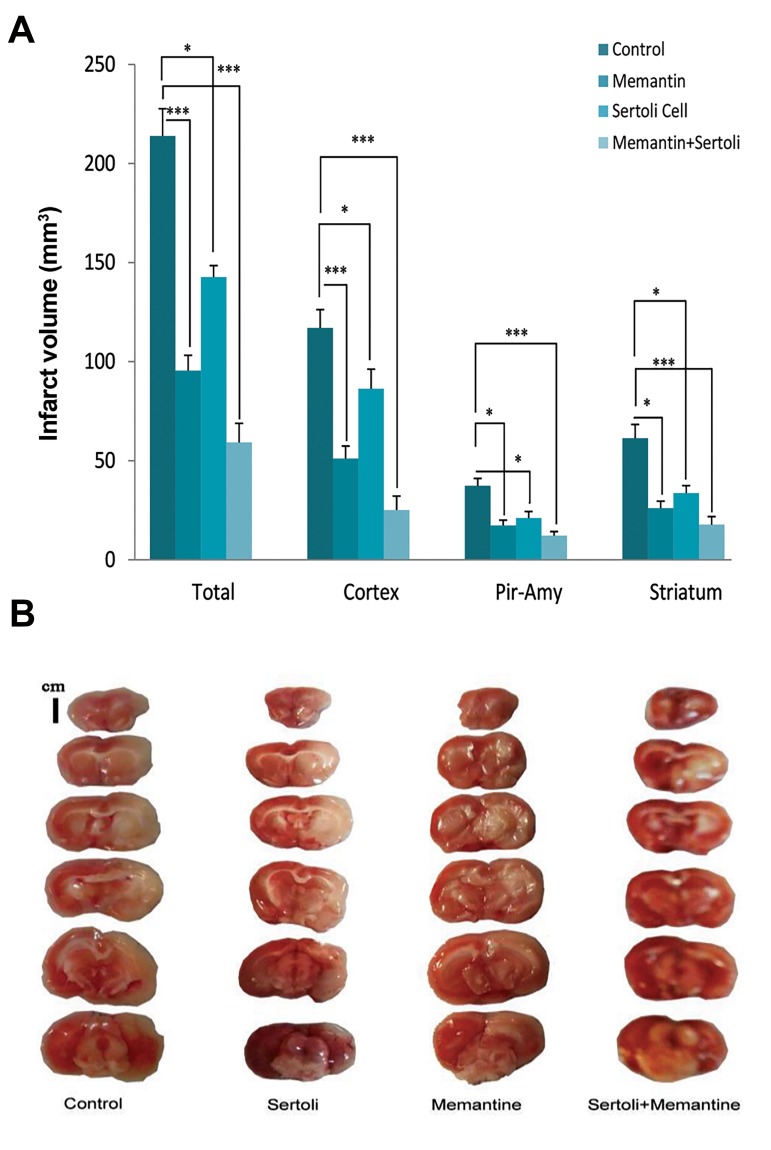
The effect of the Sertoli cell transplantation on infarction volume. **A.** The graph shows the effect of Sertoli cell transplantation, memantine, and allograft transplantation of Sertoli cells plus memantine on infarction volume in the total, cortex, striatum, and Piriform cortex-amygdala (Pir-Amy). Each column illustrates the mean ± SEM of the infarct volume (n=7). P˂0.05 compared with the control group (One-way ANOVA test). *; P<0.05 and ***; P<0.001. **B.** The Sertoli cell transplantation and injection of memantine reduced infarct volume in comparison with the control. Each column displays coronal sections of the control, Sertoli cell transplantation, memantine, and the Sertoli cells+memantine groups. Strained or red parts, as well as unstained or white parts of the brain tissue, are considered as normal and damaged areas, respectively.

The effect of SCs and memantine on infarction volume was evaluated 24 hours after reperfusion. The results showed that pretreatment of allograft SCs, memantine, and allograft transplantation of SCs plus memantine caused a reduction in infarct volume in the total, the striatum, the cortex, and the Pir-Amy regions compared with the control group (Fig .3). Analysis of the total infarct volume in the experimental groups revealed that the memantine (95.48 ± 7.70 mm^3^, P=0.001), allograft SC transplantation (142.69 ± 5.74 mm^3^, P=0.03), SC transplantation plus memantine (59.24 ± 9.59 mm^3^, P=0.001) groups indicated a significant reduction in comparison with the control group (213.86 ± 13.42 mm^3^). The administration of memantine (51.11 ± 6.31 mm^3^, P=0.003), allograft SCs (86.38 ± 9.80 mm3, P=0.03), and SC transplantation plus memantine (25.09 ± 7.40 mm^3^, P=0.001) caused a decrease in the cortex infarct volume in comparison with the control group (116.98 ± 9.26 mm^3^). The Pir-Amy infarct volume in the memantine (17.32 ± 2.69 mm3, P=0.01), the allograft SC transplantation (21.05 ± 3.32 mm^3^, P=0.04), and SC transplantation plus memantine (12.21 ± 2.03 mm^3^, P=0.001) groups was diminished compared with the control group (37.34 ± 3.63 mm^3^). Furthermore, the infarct volume of the striatum in the memantine (26.07± 3.52 mm^3^, P=0.02), the allograft SC transplantation (33.64 ± 3.80 mm^3^, P=0.04), and SC transplantation plus memantine (17.75 ± 4.04 mm^3^, P=0.006) groups showed a remarkable decrease in comparison with the control group (61.37 ± 6.95 mm^3^).

### The effect of Sertoli cell transplantation and injection of memantine in brain water content

**Fig 4:**
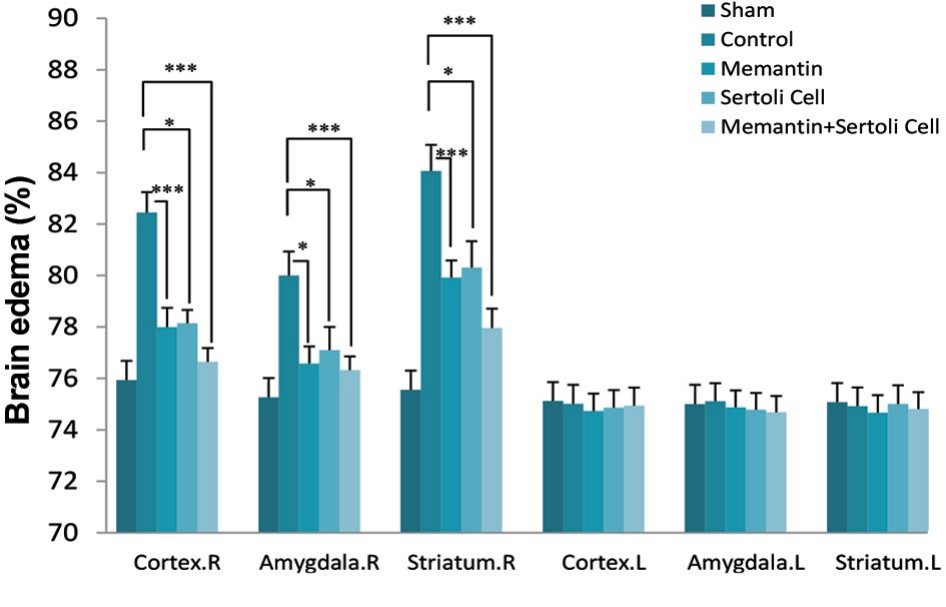
Brain edema in experimental groups, including ischemic hemisphere (Total. R), non-ischemic hemisphere (Total. L), striatum (right and left), cortex (right and left), and Piriform cortex-amygdala (Pir-Amy) (right and left) areas of the control, sham, Sertoli cells transplant, memantine, and allograft Sertoli cell transplantation plus memantine groups. Values are expressed as the mean ± SEM (n=7). P˂0.05 (One-way ANOVA test). *; P<0.05 and ***; P<0.001.

The results of brain edema also revealed the reduction of brain edema in the right striatum, cortex, and Pir-Amy regions of the brain compared with the control group (Fig .4). The beneficial effect of SC transplantation and the injection of memantine on brain edema in the cortex of the memantine- (77.99 ± 0.75%, P=0.005), allograft SC transplantation (78.14 ± 0.52%, P=0.03), and SC transplantation plus memantine (76.64 ± 0.65%, P=0.003) groups was demonstrated in comparison with the control group (82.45 ± % 0.79). Brain edema in the Pir-Amy of the memantine (76.58 ± 0.66%, P=0.01), allograft SC transplantation (77.10 ± 0.9%, P=0.01), and SC transplantation plus memantine (76.33 ± 0.53%, P=0.004) groups were minimized compared with the control group (80 ± 0.93%). The memantine (79.92 ± 0.67%, P=0.004), allograft SC transplantation (80.31 ± 1.02%, P=0.04), and SC transplantation plus memantine (77.96 ± 0.75%, P=0.001) groups showed the protective role of our therapeutic strategy (memantine and SC) on the brain water in comparison with the control group (84.18 ± 1.02%). Moreover, the brain edema was enhanced in the control group compared with the sham group in three areas of the brain, as expected (data not shown).

### The effect of Sertoli cell transplantation and injection of memantine on blood-brain barrier permeability

**Fig 5:**
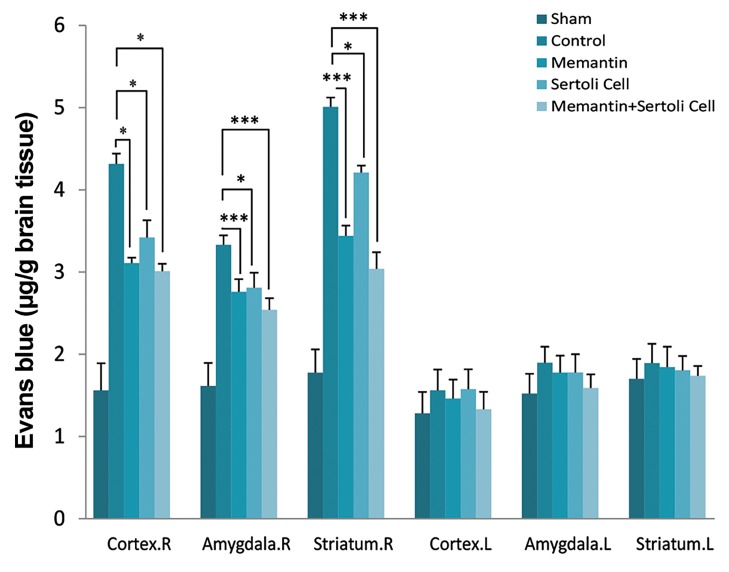
The Evans Blue extravasations in ischemic hemisphere (Total. R), non-ischemic hemisphere (Total. L), cortex (right and left), striatum (right and left), Piriform cortex-amygdala (Pir-Amy) (right and left) areas of the control, shame, Sertoli cell transplantation, memantine, and allograft Sertoli cell transplantation plus memantine groups. Values are expressed as the mean ± SEM (n=7). P<0.05 compared with the control group (Oneway ANOVA test). *; P<0.05 and ***; P<0.001.

These results showed a reduction in the BBB breakdown in the right striatum, cortex, and Pir-Amy areas in comparison with the control group (Fig .5). The BBB breakdown in the right cortex of the memantine (3.11 ± 0.06, P=0.02), allograft SC transplantation (3.42 ± 0.21, P=0.03), and SC transplantation plus memantine (3.01 ± 0.09, P=0.01) groups was decreased in comparison with the control group (4.31 ± 0.12). The reduction of BBB permeability in the Pir-Amy of memantine (2.76 ± 0.16, P=0.002), the allograft SC transplantation (2.81 ± 0.18, P=0.04), and transplantation of SCs plus memantine (2.54 ± 0.10, P=0.003) was observed compared with the control group (3.33 ± 0.28). Analysis of Evans blue concentration in the striatum of the memantine (3.44 ± 0.12, P=0.001), allograft SC transplantation (4.21 ± 0.08, P=0.02), and SC transplantation plus memantine (3.04 ± 0.20, P=0.001) groups illustrated the reduction of BBB permeability in comparison with the control group (5.01 ± 0.11). Furthermore, the increase in the BBB permeability was observed in the control group compared with the sham group in three areas of the brain, as expected (data not shown).

## Discussion

As a pioneer, we focused on investigating the effect of the combination of SC and memantine on ischemic damages. The result of the study demonstrated that allograft transplantation of SC, the injection of memantine, and simultaneous administration of SCs plus memantine reduced ischemic damages in ischemic rats. Ischemic injuries occur in forms of neurological deficits, infarction, cerebral edema, and increased permeability of the bloodbrain barrier. Transplanted SCs, along with memantine injection, significantly ameliorated these injuries in the striatum (transplantation area), cortex, and Piriform cortex-amygdala.

Evaluating sensory and motor behaviors in stroke-related research are conventional methods to identify the severity of the injury or the recovery process after treatment. The present result shows that memantine could decrease neurological deficits. According to the previous studies, the significant effect of memantine on the improvement of the neurological deficits in experimental and clinical observations was reported (11, 25, 26). With reference to the reported findings, transplantation of SCs reduced the severity of neurological function (18). For the first time, the notable reduction was also observed in the neurological deficits scores in the SCs+memantine recipient group. Probably, improvement of the neurological function is associated with attenuation of damages in related brain areas.

The other considerable result of this work is the decrease of infarction volume in both memantine-treated and SCtransplanted groups compared with the control group. Based on the preliminary reports, the positive effect of memantine on infarction volume was confirmed (11, 25, 27). In the past investigation, the SC transplantation could result in a significant reduction in infarction (18). Subsequently, the simultaneous use of transplanted SCs and memantine exerted more neuroprotective effect than the other two groups. In line with this finding, there is no report. A direct relationship was proved between infarction and cellular death (28). One of the pathophysiological processes in neurodegenerative diseases, such as cerebral ischemia, is excitotoxicity that causes severe and destructive damages.

Excitotoxicity is also defined by increased NMDA activity, glutamate level, and intracellular calcium. The extracellular glutamate levels have a direct effect on cellular death. Memantine, as a NMDA receptor antagonist, exclusively protects neurons against glutamate-induced neurotoxicity and reduces calcium intake. Consequently, it is expectable that memantine prevents excitotoxicity-related cellular loss (8). With regard to the mentioned properties of memantine, the previous report showed that the reduction of glutamate levels in the cortex, hippocampus, and striatum could be exerted following the pre-ischemic use of memantine (29). Cell therapy has attracted much attention since it could be used for the compensation of damages caused by cerebral ischemia. Transplanted SCs induce their efficacy via the improvement in ischemic damages, through various mechanisms. SCs can also be able to secrete factors that protect the brain against the injuries induced by ischemia, including GDNF and VEGF (15). It should also be stated that the increased expression of GNDF and VEGF attenuated the infarction in cerebral ischemia (30, 31).

On the other hand, the elevation of GDNF and VEGF was demonstrated by the injection of memantine in the striatum and cortex. Moreover, memantine stimulated capillary formation around the infarct area (11). Eventually, the neuroprotective effect of the combination of SCs and memantine probably was enhanced through the secretion of GDNF and VEGF.

It is worth noting that memantine and transplanted SCs could induce a remarkable effect on reducing BBB breakdown and brain edema. Similarly, other investigations confirmed the protective effect of memantine on these ischemic injuries (27, 32). Only Milanizadeh and colleagues studied the protective effect of SCs on the brain water content and permeability of the BBB in cerebral ischemia (18). On the other hand, memantine, by blocking the NMDA receptor, reduces the expression of matrix metalloproteinases (MMPs), triggering the breakdown of the BBB (33). During ischemia, the NOS enzyme increases the production of oxygen free radicals (such as ROS), impairs the cellular energy system, and finally induces DNA damage (34). Considering the antioxidant properties of memantine, as inhibitory agent against NOS activity, it can be useful in maintaining BBB integrity, as well as reducing the cerebral edema (35). The distinguished neuroprotective mechanisms of memantine are the attenuation of inflammation through decreasing activated microglia and inhibition of MAPK p38 (and NF-kB) activity (32, 36). Referring to the detrimental role of inflammation in BBB breakdown and induction of edema, memantine can prevent these damages following stroke. Oxidative stress is a pathophysiological process that happens by an imbalance in the amount of free radicals and antioxidants. So, when oxidative stress is inhibited, free radicals are reduced, and eventually, cell death is oppressed. In this respect, it was reported that oxidative stress increases the secretion of inflammatory factors, such as IL-6, IL-5, and TNF-α, triggering the breakdown of the BBB integrity and edema (37). SCs can protect the BBB through the secretion of antioxidant enzymes, including superoxide dismutase (SOD), glutathione transferase (GT), and glutathione reductase (GR). Hence, the ability of these cells in the inhibition of vasogenic edema by preventing permeability of BBB is expected (38). Furthermore, SCs can cause detrimental effects on inflammatory factors, including TNF-a, that could result in a decrease in permeability of the BBB and brain water content (39). Accordingly, the protective effect of the administration of SC plus memantine on the BBB permeability and edema is probably associated with the exclusive capabilities in the suppression of free radicals and inflammatory factors.

Referring to the previous study, the combination of the drug and cell therapy in cerebral ischemia treatment (40) can open up a new horizon for clinical research.

## Conclusion

According to the observed results in this study, the administration of memantine or SCs caused a reduction in ischemic injuries. Furthermore, the neuroprotective findings were apparent in the transplanted SCs plus memantine group. The neuroprotective effects of SCs and memantine are probably mediated by the blockage of NMDA receptor, decreasing intracellular calcium, the release of antioxidant enzymes, secretion of growth and neurotrophic factors, reducing inflammatory factors, and inhibiting apoptosis.
